# Amyloid structures: much more than just a cross-β fold

**DOI:** 10.1016/j.sbi.2019.09.001

**Published:** 2019-11-01

**Authors:** Rodrigo Gallardo, Neil A Ranson, Sheena E Radford

**Affiliations:** Astbury Centre for Structural Molecular Biology, School of Molecular and Cellular Biology, University of Leeds, Leeds, LS2 9JT, UK

## Abstract

In recent years our understanding of amyloid structure has been revolutionised by innovations in cryo-electron microscopy, electron diffraction and solid-state NMR. These techniques have yielded high-resolution structures of fibrils isolated from patients with neurodegenerative disease, as well as those formed from amyloidogenic proteins *in vitro*. The results not only show the expected cross-β amyloid structure, but also reveal that the amyloid fold is unexpectedly diverse and complex. Here, we discuss this diversity, highlighting dynamic regions, ligand binding motifs, cavities, non-protein components, and structural polymorphism. Collectively, these variations combine to allow the generic amyloid fold to be realised in three dimensions in different ways, and this diversity may be related to the roles of fibrils in disease.

## Introduction

**Figure 1:**
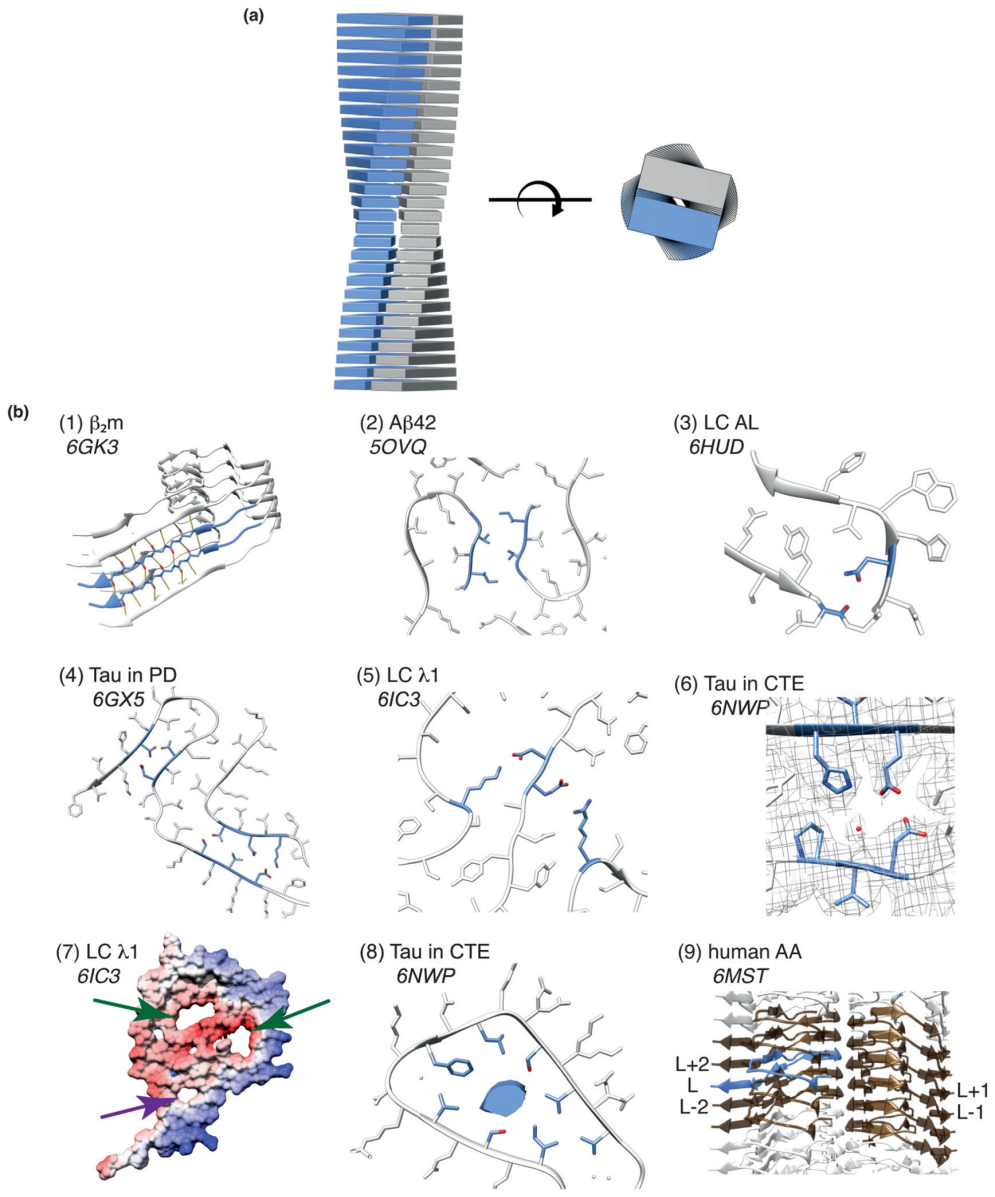
Updating the amyloid fold. **(a)** Schematic of the cross-β fold viewed from (1) side and (2) cross-section, representing the stacking of molecular layers perpendicular to the long axes of the amyloid fibrils. **(b)** Detail of the structural elements observed in amyloid cores highlighting (1) in-register parallel β-sheets [30•], (2) ‘dry’ steric zippers [29], (3) sidechain-mainchain loop hydrogen bonding [37], (4) polar zippers [26•], (5) buried salt-bridges [36••], (6) structured solvent molecules [27••], (7) polar and apolar channels (green and purple arrows, respectively) [36••], (8) cofactors (blue density) [27••] and (9) multi-molecular layer interactions between central layer ‘L’ (blue) and the adjacent layers above and below (gold) [35].

Amyloid is a conformational state that can be achieved by most (if not all) proteins [1••]. Protein sequences harbor the information necessary to enable them to fold into their native, functional 3D structures [2] or, for intrinsically disordered proteins (IDP), to remain dynamically unstructured [3]. However, proteins also contain sequences capable of forming an alternative structure(s) known as the ‘amyloid fold’ [4]. Upon cellular or physical stress, by a mechanism that is kinetically complex [5] and difficult to characterise structurally [6], one or more of these ‘amyloid-prone regions (APR)’, can rearrange to form β-strands, which stack in layers oriented perpendicular to the fibril’s long axis to generate the ‘amyloid fold’ (Figure 1a) [1••]. These β-strands and their interconnecting loops constitute the ‘amyloid core’. The repeating nature of amyloid cores, involving extensive mainchain hydrogen bonding between adjacent β-strands within the stacked layers (Figure 1b1), and close interdigitation of sidechains (Figure 1b2), results in a fibril structure that is enormously stable both thermodynamically [7] and mechanically [8]. Indeed this stability can far exceed that of the original native fold of the protein, highlighting the physico-chemical knife-edge of cellular life because of the metastable nature of their proteomes [7]. Fascinatingly, despite their high stability, fibrils are dynamic, with monomers and/or oligomers dissociating from their ends [9•,^10^], while the surface of the fibrils can act as a potent site for secondary nucleation, catalysing the formation of oligomers and new fibrils [11•,^12^].

The molecular mechanism(s) by which IDPs and initially folded amyloidogenic precursors rearrange into an amyloid core structure and stack into molecular layers is not well understood. However, it is generally accepted that this feat is accomplished via the formation of transient non-native monomeric and oligomeric species [6,13]. The transient and dynamic nature of such species has limited the characterisation of their structures and our understanding of the molecular basis of the cytotoxicity often associated to amyloid formation [14,15].

Here we review recent advances in our understanding of the amyloid fold. We describe the interactions that create the fibril core, as well as less well-ordered and dynamic regions of the amyloid fold. We also discuss differences between fibril structures formed *in vitro* and *in vivo*, and how structural polymorphism may rationalise disease phenotype. Finally, we highlight the need to combine information from multiple structural, biophysical, and cellular techniques, including information gained from *in vitro* and *in vivo* analyses, to understand amyloid formation and disease.

## Updating our understanding of the amyloid fold

### One sequence, many structures

The first high resolution structural information about amyloid came from fibrils assembled from synthetic peptides [16–19]. These structures revealed the first atomic resolution details of the stacking of β-strands in the cross-β architecture, which had been visualised at lower resolution some 50 years before [20]. The first structures of amyloid fibrils from intact proteins at atomic/near-atomic resolution were obtained from fibrils assembled *in vitr*o of the amyloi-dogenic IDPs Aβ_40_, Aβ_42_, amylin and α-synuclein using solid state NMR (ssNMR) [21–25]. The recent revolution in cryo-EM has now expanded this repertoire to fibrils formed *in vitro* or extracted *ex vivo* from β_2_-microglobulin (β_2_m), mouse and human serum Amyloid A (AA), Light Chain (LC) amyloid from two human variants, and the IDPs Tau, α-synuclein and Aβ_42_, [26•,^27^••,^28^•,^29^,^30^•,^31^–^33^, ^34^••,^35^,^36^••,^37^]. These results show that the same protein sequence can adopt different amyloid structures, leading to more fibril structures than sequences (45 amyloid fibril structures from full proteins and IDPs are currently deposited in the pdb). For α-synuclein, four distinct polymorphs have been solved by cryo-EM [31,32,33,34••] and one structure of a single protofibril by ssNMR [23]. For Tau three different neurodegenerative diseases result in fibrils with distinct, disease-specific structures, all of which are different from the structures formed *in vitro* in the presence of heparin [26•,^27^••,^28^•,^38^]. Whether this difference between *in vitro* and *in vivo* fibril structures is found for other amyloid proteins remains an important, open question.

### A convoluted amyloid fold

The structures of amyloid fibrils solved by ssNMR [21–24] and cryo-EM [26•,^27^••,^28^•,^29^,^30^•,^31^–^33^,^34^••,^35^,^36^••,^37^] have revealed that a wide variety of interactions can stabilise an amyloid core. While all of these structures have canonical cross-β amyloid folds, their structures are more complicated and diverse than originally anticipated. For amyloid precursors that are initially folded, assembly into the amyloid core requires wholesale rearrangement of the polypeptide chain and sometimes reassignment of the secondary structure elements [36••]. As expected, extensive backbone hydrogen bonding between β-strands is observed in these new fibril structures (Figure 1b1), but the topologies of the structural elements that comprise the amyloid core is more complex than those seen in fibrils formed from short peptide fragments [16,18,39,40]. The β-sheets are not, in general, formed from homotypic ‘dry steric zipper’ interactions in which two copies of the same sequence form sidechain-sidechain interactions between the β-sheets [18,41]. Such zippers are observed in fibrils formed from intact proteins, but in the interfaces formed between protofilaments (Figure 1b2) [28•,^29^,^32^]. The current list of interactions and structural motifs known to stabilise the amyloid core (Figure 1b) also includes sidechain-mainchain and sidechain-sidechain hydrogen bonding from loops that interconnect β-strands (Figure 1b3), tight interdigitation of polar and charged side chains (named here ‘polar zippers’) (Figure 1b4), buried salt bridges (Figure 1b5), interactions with solvent (Figure 1b6) [27••], and both polar [36••] and apolar [27••,^31^,^36^••] internal channels (Figure 1b7). In the latter case, an un-assigned electron density inside the apolar channel of tau filaments isolated from patiens that suffer chronic traumatic encephalopathy suggests that non-proteinaceous aliphatic molecules may participate in this amyloid core (Figure 1b8) [27••]. Finally, a given molecular layer (L), may make interactions with the layer above (L + 1), below (L − 1), oreven beyondits immediate neighbours (L + 2, L − 2, etc). Such interactions also stabilise the amyloid core (Figure 1b9) [35].

### Different forms of fibril polymorphism

**Figure 2:**
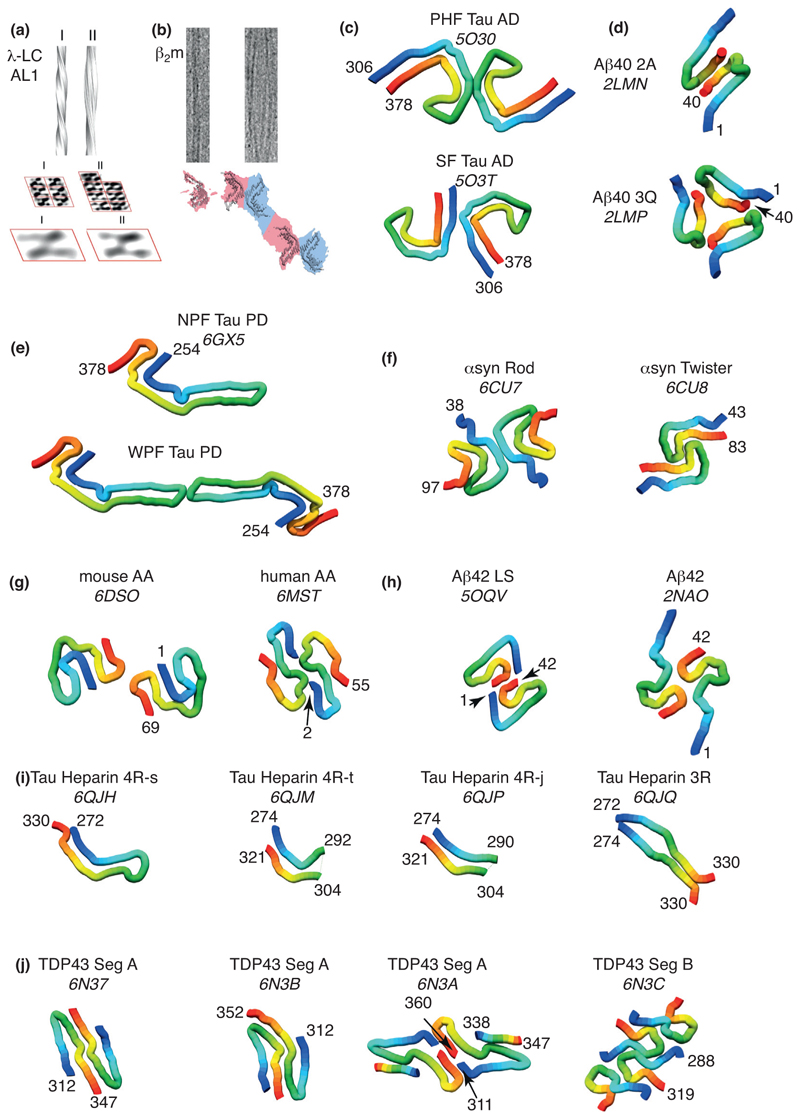
The many faces of an amyloid. Fibril polymorphs observed for **(a)** peptide fragments of LC λ [46] and for the full-length proteins **(b)** β_2_m [30•], **(c)** Tau in AD [28•], **(d)** Aβ_40_ [19,22], **(e)** Tau in Pick’s Disease [26•], **(f)** α-synuclein [32], **(g)** mouse and human serum AA [35], **(h)** Aβ_42_ [24,29], **(i**) Tau in presence of heparin [38] and **(j)** segments of TDP43 [48]. These fibrils were isolated *ex vivo* (c,e,g) or formed *in vitro* (a,b,d,f,h,i,j). Illustrations in (a) and (b) are reproduced with permission [30•,^46^].

The biological relevance of amyloid polymorphism has been extensively documented for several amyloid diseases [1••]. Fibrils created from the same precursor have been shown to display different structures in different diseases [26•,^27^••,^28^•], different seeding characteristics [42], different rates of spread [43], and distinct patterns of neuropathology [44,45]. Cryo-EM has a major advantage for structural characterisation of fibril polymorphs since each structure can (in principle) be determined independently for each co-populating polymorph, as long as sufficient images of each type can be obtained. Polymorphism can take different forms. Firstly (type 1), it can involve different packing arrangements of the same protofibril, as was observed for amyloidogenic peptides of transthyretin (TTR) [40] and immunoglobulin LC λ-1 (Figure 2a) [46], as well as for entire proteins such as β_2_m (Figure 2b) [30•]. In some cases, this type of polymorphism can involve subtle changes in the contact angle or arrangement of interactions between protofilaments, exemplified by the difference in Paired Helical Filaments (PHF) and Straight Filaments (SF) of Tau fibrils analysed from Alzheimer’s patients (Figure 2c) [28•], and in early models of fibril structures of Aβ_40_ observed by ssNMR (Figure 2d) [19,22]. In other cases, this polymorphism can involve fibrils comprised of different numbers of protofilaments, such as in the narrow (NPF) and wide (WPF) filaments observed in Tau fibrils from Pick’s disease (Figure 2e) [26•]. A second type of polymorphism (type 2) can occur when a common structure is adopted by one region of a protein sequence, while different structures are adopted by other regions. This is observed in the ‘Rod’ and ‘Twister’ polymorphs of α-synuclein fibrils (Figure 2f) [32] and in Serum Amyloid A (AA) fibrils [35] (Figure 2g). A third type of polymorphism (type 3) combines types 1 (different packing of protofilaments) and type 2 (partial common fold) and has been observed for Aβ_42_ in structures elucidated by cryo-EM [29] and ssNMR [24]. These structures exhibit a common fold (the ‘S motif’) that packs through different interfaces and with different structures for the N-terminal domain (Figure 2h). Other polymorphs of Aβ_42_ have also been observed in which different numbers of protofilaments with different twists are involved [47], but it is not yet known which molecular interactions create these different polymorphs. The fourth (type 4), and most drastic, kind of polymorphism occurs when both the protofilament structure and packing interactions vary, as observed in polymorphs of Tau fibrils formed *in vitro* in the presence of heparin (Figure 2i) [38], and in fibrils formed from fragments of TDP43 *in vitro*, in which three polymorphs are observed for the same sequence segment (Figure 2j) [48]. At least for Tau, samples from 17 patients that suffered from two variants of the same disease possessed fibrils with a similar fold, suggesting that a common fibril structure could be associated with a particular disease [49]. Yet, in all of the above cases less abundant polymorphs could also be present, albeit in too low a number to enable structure determination.

What drives fibril diversity is unclear. It could result from the intrinsic properties of the polypeptide sequence; the presence or absence of post-translational modifications; interaction with cofactors or cellular components; the nature of the environment (pH, ionic strength etc), or the cell type in which amyloid is formed. That interaction with cofactors can modulate the abundance of polymorphs has been shown for Tau, with heparin inducing structurally heterogeneous fibrils, while RNA induces structurally homogeneous fibrils [50••]. Sequence variation can also contribute to polymorphism. For example, fibrils generated from the 3R isoform of Tau in Pick’s disease (NPF and WPF, Figure 2e) [26•] are different to those generated by the 4R isoform in Alzheimer’s disease (HPF and SF, Figure 2c) [28•].

### Dynamic regions are integral to the amyloid fold

**Figure 3:**
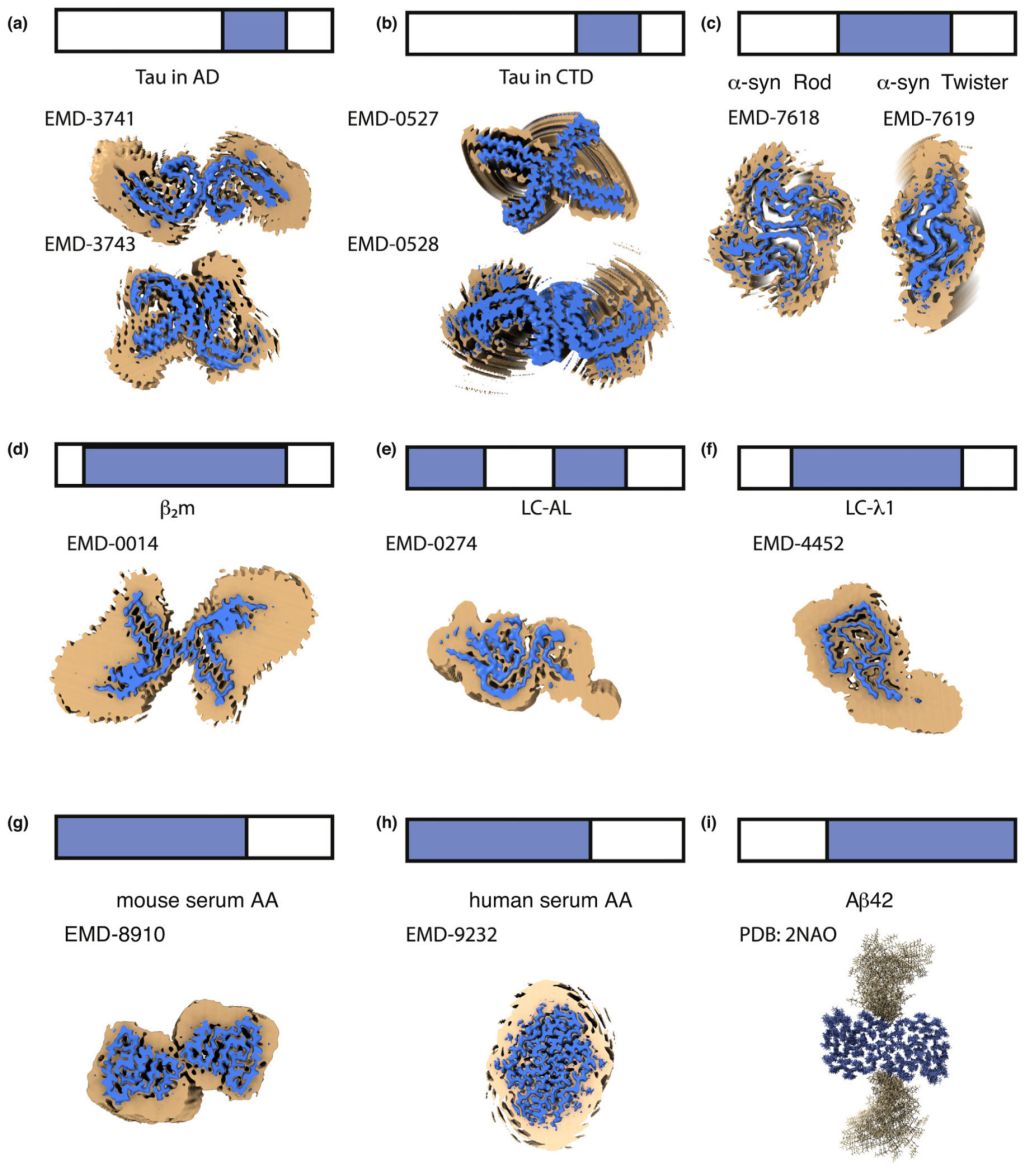
Amyloid is more than a rigid core. LDRs are shown on the structures of amyloid fibrils of full-length proteins formed *in vitro* or isolated *ex vivo*. The top of each panel shows a schematic of the full-length sequence (bar) with the sequence involved in forming the ordered amyloid core in blue and LDRs in white. The image on panels **(a−h)** corresponds to an orthogonal view down the fibril axis of the reported density maps contoured at two different levels. The regions of localised disorder are shown as broad/noisy density (orange) surrounding the amyloid core density (blue) for (a) Tau in Alzheimer’s disease [28•], (b) Tau in CTE [27••], (c) two polymorphs of α-synuclein [32], (d) β_2_m [30•], (e) *IGLV6-57* derived LC amyloid [37], (f) *GLV1-44* derived LC amyloid [36••], (g) mouse AA and (h) human AA [35]. Panel (i) shows the structure of Aβ_42_ fibrils determined by ssNMR [24], where regions of disorder are modeled as loops that point away from the core. The EMD code of each map used is indicated on each panel. The blue maps are countered to the recommended level indicated for the deposited maps. The orange maps are 5 Å low-pass filtered of the deposited map countered to 1.75 RMS using ChimeraX [72].

Another remarkable characteristic of the amyloid fibril structures determined recently is that relatively short regions of a protein sequence form the amyloid core [26•,^27^••,^28^•,^30^•,^31^,^32^,^33^,^35^,^36^••,^37^], with the remaining segments exhibiting high structural variability (Figure 3). Disordered regions map to the termini (Figure 3a−i) [23,24,26•,^27^••,^28^•,^32^], and to internal loops/segments of the polypeptide chain (Figure 3e) [37]. For example, the amyloid core of Tau fibrils involves between 72 and 94 of its 441 residues, with the number of ordered amino acids depending on the tauopathy (Figure 3a−b) [26•,^27^••]. For α-synuclein, 40−59 of its 140 residues form the amyloid core depending on the polymorph (Figure 3c) [32]. Similar observations have been made for β_2_m (63 out of 100 residues [30•] (Figure 3d), antibody LC case 1 (77 out of 111 residues [37] (Figure 3e)), or case 2 (91 out of 118 residues [36••] (Figure 3f)), SAA from mouse (69 out of 83 residues), and SAA from humans (53 out of 67 residues) [35] (Figure 3g and h, respectively). We refer to these as ‘Locally Disordered Regions’ (LDRs) to signify their localised high structural variability. LDRs have also been described in fibrils of Aβ_42_, where the N-terminal 14−15 residues, that coincide with the least amyloidogenic regions, are not involved in the amyloid core (Figure 3i) [24]. Similarly, the N-terminal 10 residues of β_2_m are highly dynamic, with the succeeding 10 residues less so (but not organised into the amyloid core) [30•]. Finally, for the structure of α-synuclein fibrils determined using ssNMR, three regions (1-24, 55-62 and 97-140) lack assignment [23] and this is usually interpreted as signifying dynamic behaviour.

LDRs are important in amyloid formation and in disease. For example, they can kinetically define the amyloid structures that result from aggregation [51]. Fibrils, including their LDRs, are also known to be involved in engaging with cellular components that regulate the health of the cell, including molecular chaperones [52], other proteins that contain IDRs or IDPs [53], components of the extracellular matrix [54•], biological membranes of different type [55] or other cellular components [56]. These dynamic regions must not be over-looked, despite the fact that they are difficult, if not impossible, to structurally characterise using cryo-EM or ssNMR. Single-molecule FRET, hydrogen/deuterium exchange, oxidative labelling and cross-linking methods offer exciting possibilities to characterise these regions and their interactions *in vitro* and *in vivo* in the future.

### Left or right-handed, parallel or anti-parallel?

Contrary to the canonical right-handed β-sheets observed in globular proteins, amyloid fibrils can adopt right-handed or left-handed β-sheets, with a switch between handedness requiring only subtle differences in the β-strand φ/ψ angles [57]. For example, mouse and human AA amyloid have opposite chirality despite having 78% sequence identity [35]. While anti-parallel β-strands were observed in amyloidogenic fragments using X-ray crystallography [18,41], ssNMR [16] and X-ray fibre diffraction [58], amyloid fibrils formed from longer precursors commonly adopt a parallel in-register structure (Figure 1a and b1) [1••]. In these structures each molecular layer ‘L’ deviates from planarity, which allows intermolecular interactions beyond the immediate neighbouring layers ‘L + 1’ and ‘L-1’ [29,35]. The number of molecules that can interact in this mode can span up to 10 molecular layers, as observed in human AA amyloid (Figure 1b9) [35]. Non-planarity of the layers also confers a subtle polarity to the fibrils because it generates structural differences between the two fibril ends.

### Amyloid structures: beyond protein

The interactions between amyloid fibrils and cofactors has been long studied, with the list of ligands including nucleic acids [50••,^59^], lipids [60], metal ions [61], glycosaminoglycans [62], glycoproteins [63] and others (reviewed in Ref. [54•]). The consequences of these interactions include modulation of fibril growth kinetics and fibril stability [62,64], changes in amyloid-associated cytotoxicity [65] and altered seeding efficiency [50••]. The recent elucidation of the structure of Tau fibrils extracted from patients with Chronic Traumatic Encephalopaty (CTE) have provided the first atomic-resolution information showing that co-factors can be an integral part of the amyloid core (Figure 1b8) [27••].

### The amyloid fold *in vivo*

Recent developments in Cryo-Electron Tomography (CryoET) have started to reveal the organisation of amyloid fibrils *in situ* and how fibrils can disturb cellular processes [66,67•]. Using kinetic experiments *in vitro* amyloid fibril formation can be explained as a combination of elementary mechanisms including primary/secondary nucleation, elongation and fragmentation [68••]. This has allowed the identification of small molecules and antibodies/chaperones that target specific steps in amyloid assembly [69,70]. Importantly, the finding that the same compounds are active *in vitro* and *in vivo*, validates the utility of *in vitro* observations for understanding amyloid disease. In the same way, fluorescent oligothiophene conjugates designed *in vitro* have also been used to analyse amyloid *in situ* and shown to be able to differentiate conformational variants of Ab plaques in patients with different subtypes of Alzheimer’s disease [71].

**Figure 4:**
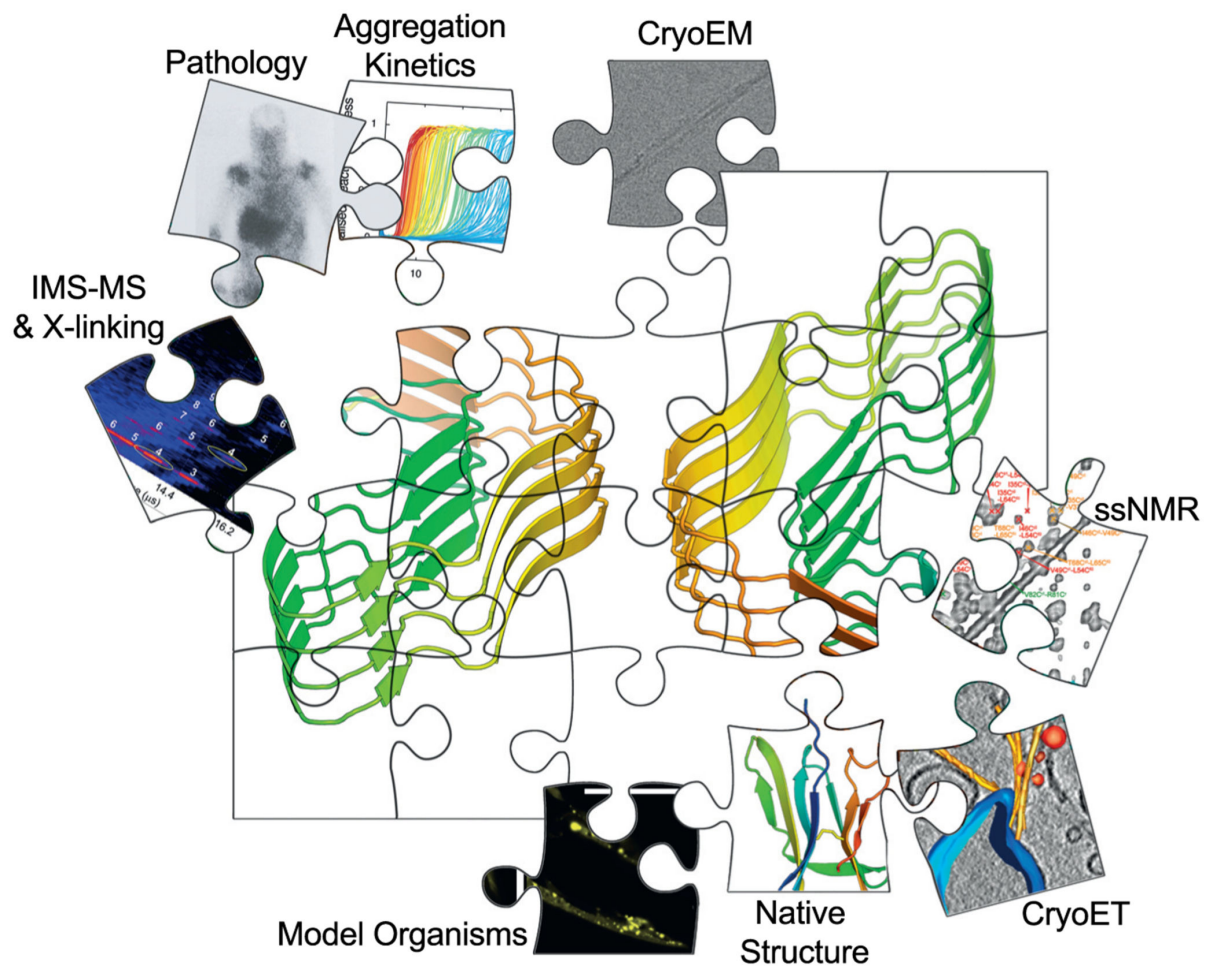
A combination of techniques is required to understand the amyloid fibril structure and its cellular consequences. However, the picture is still incomplete. The missing aspects will be achieved through biochemical, biophysical and cellular investigation. Only by an integrative approach in which *in vitro, ex vivo, in situ* and *in vivo* approaches are combined can we hope to achieve the structural, cellular and mechanistic understanding required to fully understand the amyloid structure and to inspire biomedical progress.

Although extraordinary progress has been made in our understanding of amyloid *in vivo*, the resolution currently possible by cryo-ET (~3 to 10 nm) does not enable fibril structure details to be visualised within cells. Hence, the full diversity and complexity of the amyloid fold *in vivo* are yet to be revealed. To complete the picture (Figure 4) we need to improve the resolution of fibril structures *in vitro* and *in situ*, and to employ different techniques, in combination, to inform on different aspects of the amyloid fold, including the functionally important dynamic regions discussed above. We also need to remember that oligomers also play a key role in amyloid disease [14,15], yet structurally characterising these species remains a hugely challenging task *in vitro*, and is not possible currently *in vivo*.

## Summary and outlook

Recent discoveries about the structure of amyloid fibrils have shifted our understanding of the amyloid fold from an initially simple set of structural elements, to a complex architecture in which apolar, polar, ionic and hydrogen bonding interactions, together with solvent and co-factor binding, structured cores and locally disordered regions, build the fibril. Cofactors and post-translational modifications can also have profound effects on the structure, kinetic and thermodynamic properties of amyloid fibrils and their cellular effects. By integrating methods able to interrogate the structured and dynamic regions of amyloid, and exploiting the powers of cryo-EM/ET to determine amyloid structures *in vitro, ex vivo* and *in situ*, we will soon have a better understanding of the amyloid fold as a whole and how this amazingly diverse, but stable structure, affects cells. Rather than a generic and simple amyloid fold, there is a remarkable array of amyloid structures, each of which may uniquely contribute to the generation of cellular dysfunction and disease.
